# An Iridium Complex as Bidentate Halogen Bond‐Based Anion Receptor Featuring an IncreasedOptical Response

**DOI:** 10.1002/open.202300183

**Published:** 2024-04-10

**Authors:** Robin Kampes, Avinash Chettri, Maria Sittig, Guangjun Yang, Stefan Zechel, Stephan Kupfer, Martin D. Hager, Benjamin Dietzek‐Ivanšić, Ulrich S. Schubert

**Affiliations:** ^1^ Laboratory of Organic and Macromolecular Chemistry (IOMC) Friedrich Schiller University Jena Humboldtstraße 10 07743 Jena Germany; ^2^ Jena Center for Soft Matter (JCSM) Friedrich Schiller University Jena Philosophenweg 7 07743 Jena Germany; ^3^ Leibniz Institute of Photonic Technology Jena Albert-Einstein-Straße 9 07745 Jena Germany; ^4^ Institute of Physical Chemistry Friedrich Schiller University Jena Helmholtzweg 4 07743 Jena Germany; ^5^ Center for Energy and Environmental Chemistry Jena (CEEC Jena) Friedrich Schiller University Jena Philosophenweg 7a 07743 Jena Germany

**Keywords:** Supramolecular chemistry, Halogen bond, Anions, Luminescence, Sensing

## Abstract

We present a luminescent Ir(III) complex featuring a bidentate halogen bond donor site capable of strong anion binding. The tailor‐made Ir(III)(L)_2_ moiety offers a significantly higher emission quantum yield (8.4 %) compared to previous Ir(III)‐based chemo‐sensors (2.5 %). The successful binding of chloride, bromide and acetate is demonstrated using emission titrations. These experiments reveal association constants of up to 1.6×10^5^ M^−1^. Furthermore, a new approach to evaluate the association constant by utilizing the shift of the emission was used for the first time. The experimentally observed characteristics are supported by quantum chemical simulations.

## Graphical abstract

We present a luminescent Ir(III) complex featuring strong halogen bonding of anions as a chemo‐sensor. The strong binding of anions is determined using the shift of the emission energy as a reliable readout parameter for evaluation of association constants. Those results were additionally corroborated by quantum chemical calculations.

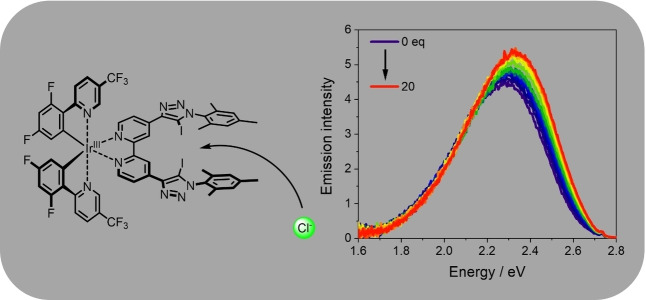

## Introduction

The halogen bond (XB) represents a supramolecular interaction between a halogen bond donor (*i. e*. Lewis acid) and a halogen bond acceptor (*i. e*. Lewis base).^[1]^ Within the XB donor moiety, the halogen atom is covalently linked to a polarizing group R. This polarization facilitates the formation of a Lewis acidic region on the halogen atom called σ‐hole.^[2]^ It is located at the opposite side of R causing the XB's preference of a highly linear arrangement. This behavior differs the XB from the hydrogen bond, which is often less impacted by deviations from the linear arrangement of the donor and acceptor.^[3]^ Moreover, the strength of the XB can be tuned in a simple manner *via* the XB donor. For that purpose, variation of the polarization strength of R and the choice of the halogen atom (I>Br>Cl) can be utilized.^[2]^ These characteristics render the XB as a powerful tool in supramolecular chemistry.[^3c^, ^4^] Hence, the XB is frequently applied in research fields like crystal engineering,^[5]^ smart polymer materials,^[6]^ organo catalysis^[7]^ as well as anion recognition and sensing.^[8]^

The importance of anion recognition chemistry is provoked by the ubiquitous role of anions in biological, chemical and environmental processes.^[9]^ XBs have shown their suitability for anion recognition due to their characteristics (as partly stated above), in particular due to the high and tunable interaction strength, the tolerance against water and the high preference for a linear arrangement.[^3c^, ^4^] This dependency on linear arrangement enables the design of more selective receptors due to geometrical restrictions with different guest sizes. Such an approach is, *e. g*. presented by the Beer group's rotaxane receptors.^[10]^ Hence, the XB‐based receptors are often superior compared to their hydrogen bond counterpart as presented in various studies.^[11]^

For the development of novel sensors, several design criteria have to be considered.^[12]^ On the one hand, the sensor must feature efficient binding of the analyte. On the other hand, a strong response *i. e*. transduction of the binding to a macroscopically and externally readable signal is crucial.^[13]^

Luminescent compounds are very suitable for such a purpose due to the simple and highly sensitive measurability *via* emission spectroscopy.^[14]^ Key examples are transition metal complexes.^[15]^ In particular, Ir(III) complexes can feature both, strong binding and high luminescence when combined with a suitable binding site. Hence, these complexes were frequently utilized as chemo‐sensors for various types of analytes.^[16]^ For anion sensing, *e. g*., hydrogen bond‐based sensors^[17]^ or XB‐based sensors^[18]^ were reported. These complexes feature a range of different phenyl pyridine‐based ligands with varying substituents and, consequently, a wide range of emission quantum yields. Within this range, the commonly utilized 2‐(4‐methylphenyl)pyridine (Meppy) results in rather low emission quantum yields compared to other motifs like (2‐(2,4‐difluorophenyl)‐5‐(trifluoromethyl)pyridine (dFCF_3_ppy), *i. e*. 0.18 *vs*. 1.00 ([Ir(Meppy)_2_(bpy)]^+^ *vs*. [Ir(dfCF_3_ppy)_2_(bpy)]^+^) relative quantum emission yield.[^16^, ^19^] To significantly increase the emission quantum yields, fluorinated ligands like 2‐(2,4‐difluorophenyl)‐5‐(trifluoromethyl)pyridine can be used.[^16^, ^19^] The observed response of the complexes to anion binding in these examples is reflected in changes of the emission intensity (*i. e*. emission quantum yield) or by monitoring a distinct shift of the emission band (*i. e*. emission energy change). To the best of our knowledge, literature examples usually utilize the emission intensity changes to determine the association constants or deviate to other methods like ^1^H NMR titrations.^[20]^

Consequently, we report a new XB‐based anion sensor (**IrF‐XB**) with a bidentate XB donor ligand and two cyclometallating dFCF_3_ppy ligands aiming for a stronger emission response of the sensor compared to previous systems (see Scheme S2). This evolution is leading to an excellent anion sensing system based on XB‐interaction. Moreover, we were able to access the association constants of this system *via* the shift of the emission maximum.

## Results and Discussion

**Scheme 1:**
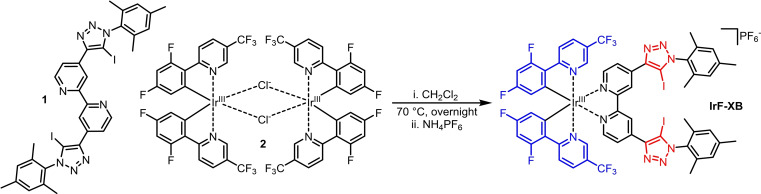
Schematic representation of the **IrF‐XB** synthesis from the XB donor ligand **1** and the binuclear Ir(III)‐precursor **2**. The triazole XB donor moieties are highlighted in red while the substituted ligands are emphasized in blue.

The facile synthesis of the complex **IrF‐XB** (see Scheme 1 and SI Scheme S1) started from ligand **1** which was prepared in a comparable manner to a previous literature report.^[18]^ In order to synthesize a new Ir(III) complex featuring an improved emission quantum yield, a different binuclear precursor **2** bearing electron withdrawing substituents on the two phenyl pyridine based ligands was utilized.^[19]^ Complexes based on those ligands typically feature higher emission quantum yields up to five times compared to Meppy. Hence, the substitution should increase the sensor's response signal. To the best of our knowledge this is the first time those ligands are used for XB‐based anion sensors. The compound **IrF‐XB** was characterized by ^1^H and ^19^F NMR spectroscopy (the spectra are displayed in the SI (Figure S1 and Figure S2)). High resolution ESI‐MS spectrometry confirmed the high purity of the resultant compound. Importantly the anion was exchanged to PF_6_^−^, a non‐binding anion as demonstrated below.

**Figure 1:**
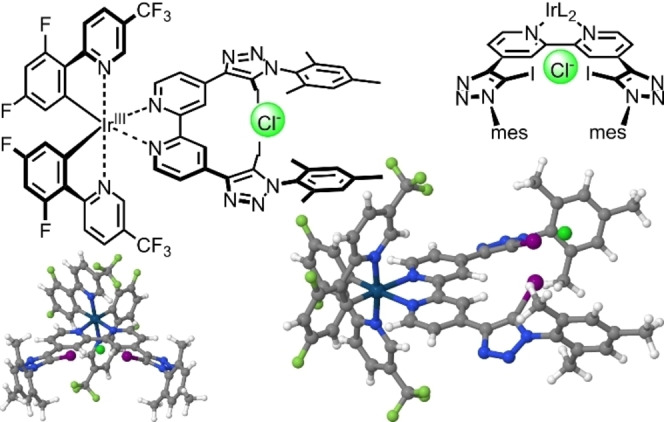
Schematic representation and DFT‐predicted structure of **IrF‐XB** and its XB mode with a chloride ion (highlighted in green) according to density functional theory^[22]^ and X‐ray data on a structurally related Ir(III) complex.^[18]^ The chloride, which is located slightly above the bipyridine plane, is bound by both iodotriazole moieties.

The Ir complex features two XB donor moieties and is, hence, capable to form two XBs. If accurately designed, this property can increase the binding strength compared to monodentate complexation by roughly two orders of magnitude as reported in literature.^[21]^ However, it also induces geometrical constraints due to the XB's high demand of linearity. Hence, we anticipate that the sensor should be best suited for smaller anions like chloride.^[18]^ The molecular structure as well as the binding mode were derived based on our literature‐reported comparable complexes^[18]^ and quantum chemical calculations^[22]^ (see SI Table S6). Both results are depicted in Figure 1. With increasing anion size the distortion displayed increases leading to a less attractive binding. In addition to the bidentate anion binding site, the sensor is positively charged, which additionally increases its interaction with anions.

We performed emission quantum yield (ϕ_em_) measurements to investigate if **IrF‐XB** is principally suitable as an optical anion sensor. As mentioned in the introduction, a strong modulation of the emission properties upon anion binding is desirable, *i. e*., a strong emission, which changes significantly in intensity (or wavelength). For the corresponding emission measurements, air saturated acetonitrile solutions were used for both the investigation of the complex as well as for the titration experiments. The choice to work in air saturated solutions has been made in view of possible applications of the molecular anion sensor, although the obtained quantum yields are smaller compared to those measured in inert solutions.^[23]^ Within these experiments **IrF‐XB** revealed an emission quantum yield of 8.4 % determined via relative emission quantum yield measurements using Coumarin 153 in ethanol as emission quantum yield standard.^[24]^ A direct comparison to other literature the reported iridium complexes is not straight forward due to different experimental conditions employed, *e. g*., the use of different conditions like air saturated or deaerated solutions. However, compared to the literature known Meppy‐based anion sensor Ir(III) complex^[18]^ (2.5 %), **IrF‐XB** reveals a more than doubled emission quantum yield.

To investigate the change of emission upon anion binding, emission titration experiments were performed and supported by quantum chemical simulations. Compared to the common evaluation of binding constants with ^1^H NMR spectroscopy, emission titration is simpler, faster and enables an investigation at very low sensor concentrations.^[25]^ Furthermore, it is more application orientated, since emission investigations can be read out more easily. In addition, the structural and electronic properties of **IrF‐XB**, **IrF‐XB×Cl**, **IrF‐XB×Br**, **IrF‐XB×Ac** and **(IrF‐XB)2×Ac** (*i.e*. the complex upon anion bonding) were unambiguously evaluated at the density and time‐dependent density functional levels of theory (DFT and TDDFT). All calculations addressing the ground state structures were performed utilizing the Gaussian16^[26]^ program. Singlet (S_0_) and triplet ground state (T_1_) equilibrium structures were obtained for all systems by DFT utilizing the B3LYP^[27]^ exchange‐correlation functional in association with the def2‐SVP^[28]^ basis set as well as the respective core potentials. To evaluate scalar relativistic effects on the various excited states within the Franck‐Condon region, TDDFT calculations were performed utilizing ORCA 5.0.2,^[29]^ *i.e*. employing the scalar relativistic zeroth‐order regular approximation (SR‐ZORA).^[30]^ Further details regarding the computational setup and results are collected in the SI.

During the titration experiments, the emission spectra of **IrF‐XB** were recorded while stepwise increasing the guest concentration. As anion source, tetrabutylammonium (TBA) salts of the halides, chloride and bromide, the oxoanion acetate and hexafluorophosphate were used. First, in a control experiment the anion PF_6_^−^ was applied. Upon addition of PF_6_^−^ to the solution of the Ir complex, neither a shift of the emission maximum nor a change of the emission quantum yield was observed. This finding can be explained with the non‐binding character of the PF_6_^−^ ions and fits well to the expectation. In contrast, *e. g*., upon addition of chloride the emission maximum shifted hypsochromically (from 543 to 531 nm; ΔE=0.05 eV), accompanied with an increase of the emission quantum yield of 20 % to reach the final value of 10.1 %. The emission quantum yield of pure **IrF‐XB×Cl^−^** was estimated to 10.2 % (for details see Figure S4).

**Figure 2:**
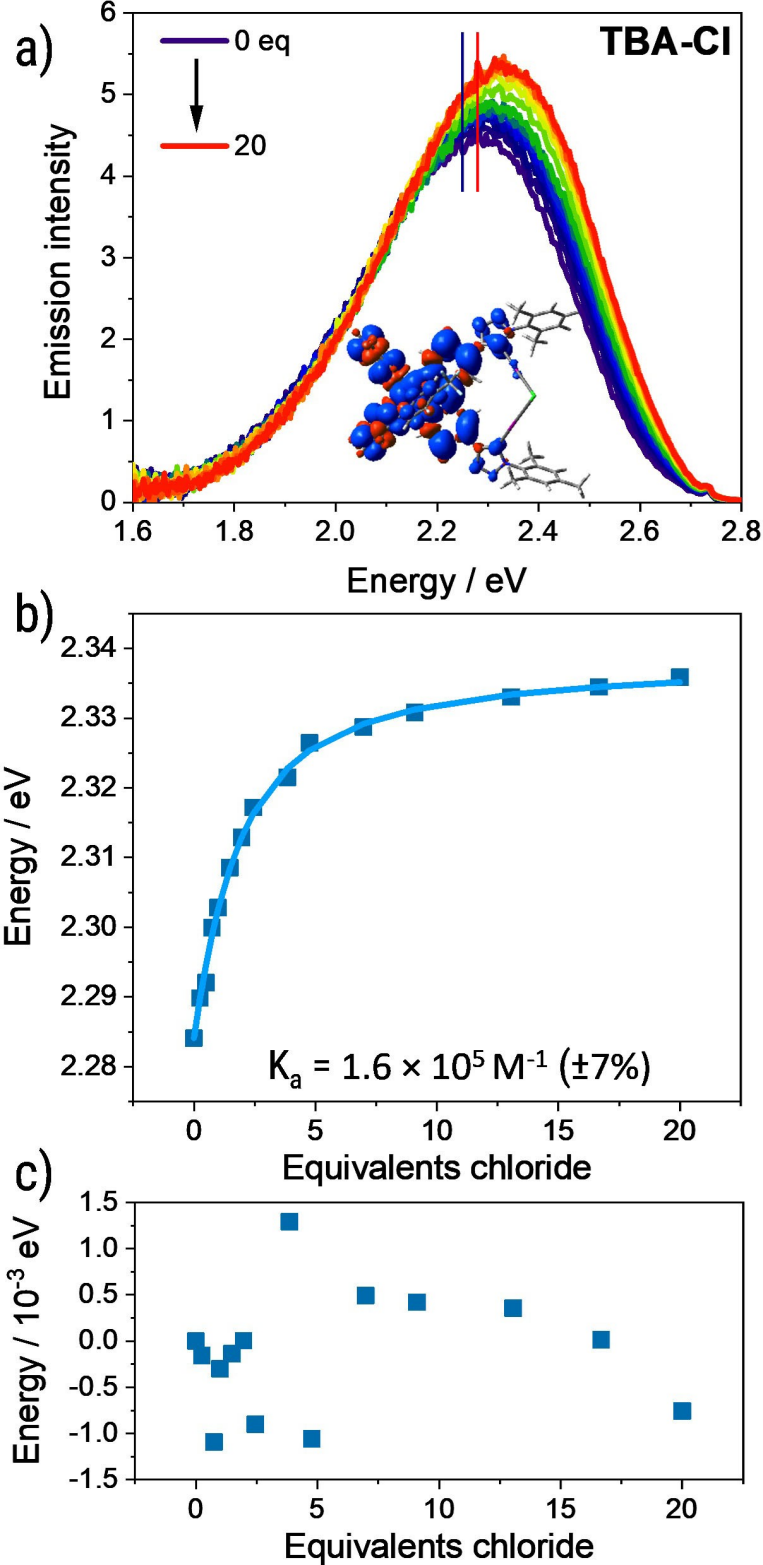
a) Emission spectra of the titration experiment of **IrF‐XB** against tetrabutylammonium chloride in acetonitrile (c_**IrF‐XB**_ 5×10^−6^ M). The observed emission band shifts to higher energy upon addition of the guest and the emission intensity increases. DFT calculated emission energies are highlighted with reference lines. Additionally, the nature of the ^3^MLCT state responsible for this emission is displayed. For detailed view see SI Figure S22; b) fit of the emission maxima in eV against the equivalents guest added (K_a_=1.6×10^5^ M^−1^±7 %) and c) residuals of the fit. The free open source program Bindfit was used for the determination of association constants.^[31]^

To evaluate the origin of the emission modulation, we performed quantum chemical simulations. The computational modelling of the phosphorescence of **IrF‐XB** and its alteration by means of XB‐coordinated Cl^−^ ions yielded emission wavelengths of 543 and 531 nm, respectively, while the hypsochromic shift (0.03 eV) is in excellent agreement with the emission data stemming from the titration experiments. In both cases (**IrF‐XB** and **IrF‐XB×Cl**), the emissive triplet state (T_1_) is of metal‐to‐ligand charge transfer (^3^MLCT) character and involves π* orbitals of all three ligands (see spin density Figure 2a) and SI Figure S22). According to scalar‐relativistic TDDFT, such low‐lying ^3^MLCT states are rapidly accessible by intersystem crossing and pronounced spin‐orbit couplings of up to 530 cm^−1^ among the singlet and triplet MLCT states (see SI Table S3–S7). Therefore, we attribute both characteristics – the hypsochromic shift as well as the modulation of the emission intensity – to the binding of the anion. Similar behavior was observed for bromide and acetate as guest ions whereas the shift was less pronounced for the same guest concentration indicating a weaker binding (compare Figure 2b), SI Figure S8 and S13 as well as Table S2). In contrast to chloride, the trend of the emission quantum yield is complicated, lacking the clear increase during the titrations. This behavior was already reported for similar complexes.^[18]^ Notably, the performed DFT simulations upon binding of a chloride anion show a pronounced planarization of the binding pocket (see Table S8). In contrast, the binding pocket is too small to fully host bromide or acetate. This might explain the different effect of anion complexation on the emission quantum yield. Consequently, we focused on the shift of the emission maximum during the binding studies for more reliable calculations as described in the following paragraph. The reason for this might be the less planar arrangement of the bound complex in terms of the bipyridine triazole plane due to steric restrictions as supported by DFT (see SI Table S6).

Subsequently, the steady state emission spectra were used to calculate the association constants. Contrary to previous studies that utilize the emission intensity,^[20]^ the emission maximum's corresponding wavelength was determined (see SI for details) and plotted against the host to guest equivalents (See Figure 2 and SI Figure S5 to Figure S20). This new method turned out to be less vulnerable to other effects influencing the emission intensity as reported previously.^[18]^ The data was subsequently fitted with the open source program Bindfit using an 1 to 1 association model.^[31]^ All obtained association constants are displayed in Table 1. The strongest binding (K_a_=1.6×10^5^ M^−1^ ± 7%) was observed for chloride ions, which is in accordance with previous observations for comparable XB binding sites.^[18]^ This finding is presumably caused by geometrical restrictions of the binding site and the higher charge density and Lewis basicity of chloride among the halides. In general, this makes chloride a better XB acceptor compared to bromide and iodide.^[11a]^ The binding of acetate and bromide is comparably strong, which is also in line with literature reports.^[18]^ In general, we observed a slight trend towards stronger binding (approximately doubled K_a_) in case of **IrF‐XB** for all studied anions compared to a previously reported Ir‐complex (see Table 1 and SI Table S1), although the conditions were slightly different.^[18]^ This result might indicate that the fluorination of the auxiliary ligands also influences the XB strength positively, *e. g*., due to a stronger polarization. However, the titrations confirmed the suitability of the fluorinated complex **IrF‐XB** to strongly bind anions as required for anion sensing.

**Table 1:** Association constants of **IrF‐XB** determined *via* emission titrations in 5×10^−6^ M acetonitrile solutions. Data fitted using the 1 to 1 association model in Bindfit.^[31]^

	**Cl^−^**	**Br^−^**	**Ac^−^**	**PF_6_** ^**−**^
**K_a_ (M^−1^)**	1.6×10^5^ ±7 %	3.6×10^4^ ±12 %	8.4×10^4^ ±14 %	–

## Conclusions

In conclusion, the fluorinated Ir(III) complex **IrF‐XB** was synthesized from binuclear precursor **2** and the established bipyridine‐based XB‐donor ligand **1**. The use of 2‐(2,4‐difluorophenyl)‐5‐(trifluoromethyl)pyridine as auxiliary ligand resulted in a high emission quantum yield of 8.4 % in air‐saturated acetonitrile solution. The complex revealed strong anion binding, which was investigated *via* emission spectroscopy titration experiments and quantum chemical simulations. The strongest binding was found for chloride with K_a_=1.6×10^5^ M^−1^ (±7 %). Hence, a slight tendency towards stronger binding within the range of investigated anions compared to the literature^[18]^ was found. Both aspects, higher emission quantum yield and stronger binding, render this complex as a very promising candidate as anion sensor. The simple measurability of the luminescence generally renders this method highly suitable for the determination of association constants under such conditions (*i. e*., low concentrations to determine high K_a_ values) compared to NMR spectroscopy. Moreover, the utilization of the emission energy shift resulted in a simple and reliable determination of the association constants. Compared to previously reported emission intensity‐based assessment of association constants, the approach is more robust against other phenomena influencing the emission intensity. The improved luminescence of our Ir(III)‐based anion sensor compared to other Ir(III)‐based anion sensors in literature can be a step towards further developments regarding optimized binding site. A possible aim is the adaption of the binding site to larger anions *i. e*., to increase the size of the receptor cavity. This adaption would ensure a planar arrangement leading to higher association constants and an emission increase for larger anions.

## Supporting Information

The authors have cited additional references within the Supporting Information.[^26^, ^27^, ^28^, ^29^, ^30^, ^32^]

## Conflict of interests

The authors declare no conflict of interest.

1

## Supporting information

As a service to our authors and readers, this journal provides supporting information supplied by the authors. Such materials are peer reviewed and may be re‐organized for online delivery, but are not copy‐edited or typeset. Technical support issues arising from supporting information (other than missing files) should be addressed to the authors.

Supporting Information

## Data Availability

The data that support the findings of this study are available in the supplementary material of this article. DFT‐optimized structures are available free of charge via Zenodo (Ref. [22], https://doi.org/10.5281/zenodo.10105169).
