# Supplementary material for: An Iridium Complex as Bidentate Halogen Bond‐Based Anion Receptor Featuring an IncreasedOptical Response
Source: ChemistryOpen. 2024 Apr 10;13(5):e202300183. doi: 10.1002/open.202300183 (PMC11095211; doi:10.1002/open.202300183)
Supplement: Supplementary file 1 — Supporting Information [file OPEN-13-e202300183-s001.pdf]

ChemistryOpen

Supporting Information

An Iridium Complex as Bidentate Halogen Bond-Based Anion Receptor Featuring an IncreasedOptical Response

Robin Kampes, Avinash Chettri, Maria Sittig, Guangjun Yang, Stefan Zechel, Stephan Kupfer, Martin D. Hager, Benjamin Dietzek-Ivanšić,* and Ulrich S. Schubert*

Supporting Information

An iridium complex as bidentate halogen bond-based anion sensor featuring an increased optical response

Robin Kampes,[a], [b] Avinash Chettri,[c], [d] Maria Sittig,[c], [d] Guangjun Yang,[d] Stefan Zechel,[a], [b] Stephan Kupfer,[d] Martin D. Hager,[a], [b], [e] Benjamin Dietzek-Ivanšić*,[b], [c], [d], [e] and Ulrich S. Schubert*[a], [b], [e]

[a] Laboratory of Organic and Macromolecular Chemistry (IOMC), Friedrich Schiller University Jena, Humboldtstraße 10, 07743 Jena, Germany

[b] Jena Center for Soft Matter (JCSM), Friedrich Schiller University Jena, Philosophenweg 7, 07743 Jena, Germany

[c] Leibniz Institute of Photonic Technology Jena e. V., Albert-Einstein-Straße 9, 07745 Jena, Germany

[d] Institute of Physical Chemistry, Friedrich Schiller University Jena, Helmholtzweg 4, 07743 Jena, Germany

[e] Center for Energy and Environmental Chemistry Jena (CEEC Jena), Friedrich Schiller University Jena, Philosophenweg 7a, 07743 Jena, Germany

Table of content:

Materials and instrumentation 2

Synthesis 3

Ir(2-(2,4-difluorophenyl)-5-(trifluoromethyl)pyridine)$_2$(**1**) – **(IrF-XB)** 3

NMR spectroscopy 5

Emission titrations of IrF-XB 6

Computational details 17

Simulated Franck-Condon photophysics and intersystem-crossing 17

References 26

Materials and instrumentation

All chemicals were bought from TCI Germany, Sigma Aldrich, Alfa Aesar, Fluorochem, Roth and Across Organics and were used as received. Though 2-(2,4-difluorophenyl)-5-(trifluoromethyl)-pyridine was purified *via* column chromatography (silica, dichloromethane) prior to use. Compounds **1**[1] and **2**[2] were synthesized (without recrystallization) as published in literature (see also **Scheme S1**). Tetrabutylammonium salts used for the titration experiments were purchased in high purity and dried in a desiccator prior to use.

NMR spectra were recorded on a 500 MHz BrukerAvance IV (NEO) spectrometer and a Bruker Fourier300 (300 MHz) spectrometer in deuterated solvents (Eurisotop) at 297.1 K and referenced by the solvent signals. HR-ESI-MS spectra were recorded on a Bruker ESI-(Q)-TOF micrOTOF QII mass spectrometer operated in positive ion mode with the standard electrospray ionization. Dichloromethane and acetonitrile were used as solvents. Column chromatography was performed on a Biotage Isolera One flash chromatography system using Macherey-Nagel Chromabond Flash BT 4 SiOH cartridges.

Steady state absorption spectra were measured in quartz cuvettes with (d = 1 cm, Hellma) on a Jasco V780 UV/Vis/NIR spectrophotometer. Steady state emission measurements were carried out on a FLS980 photoluminescence spectrometer from Edinburgh Instruments equipped with a Xe lamp (ozone free 450 W xenon bulb) as excitation source. The quantum yield estimation was carried out using Coumarin 153 (Φ_{fl} = 0.38) in EtOH[3] as emission reference standard.

Synthesis

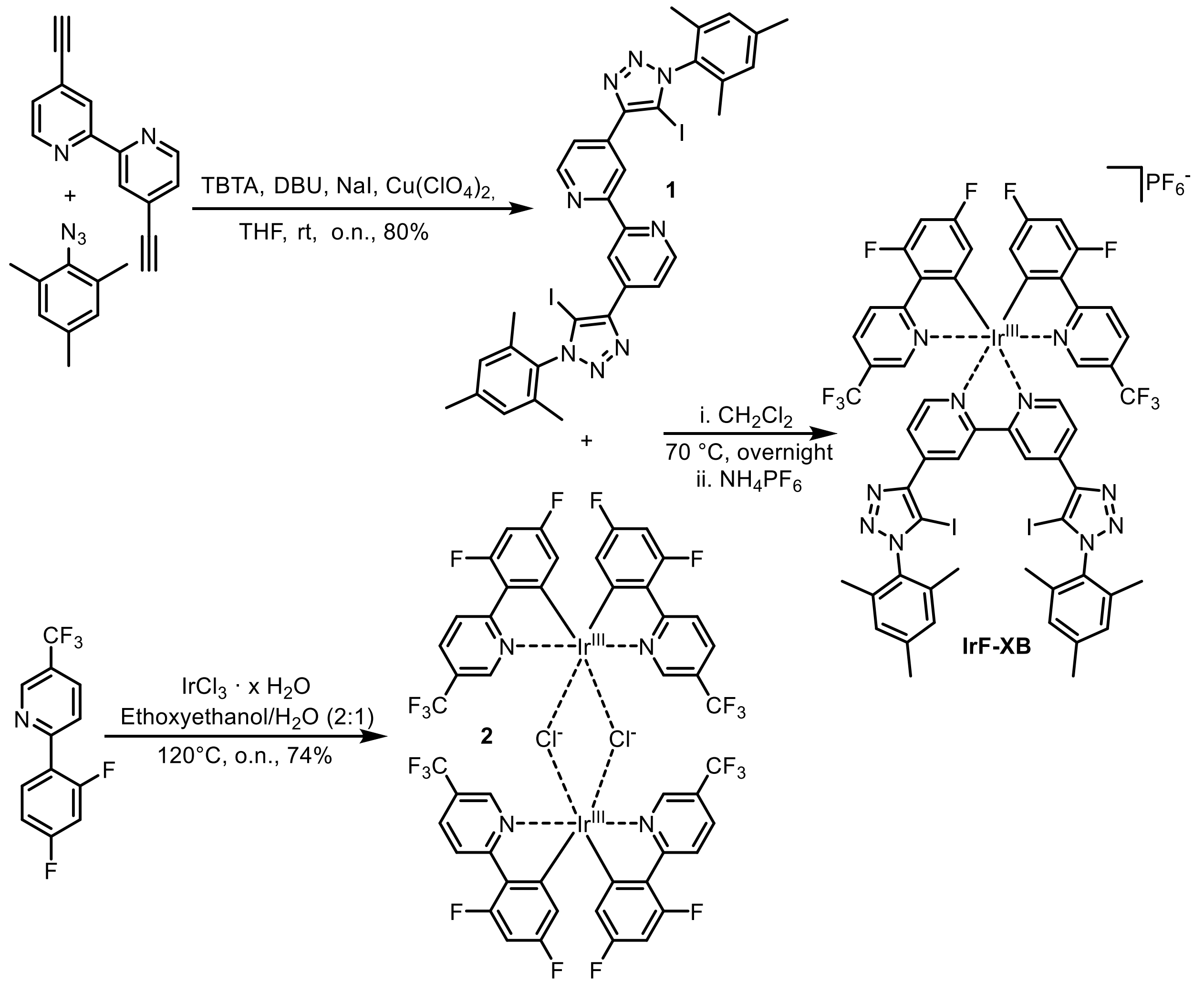

Scheme S1. Schematic representation of the synthesis of **1**, **2** and **IrF-XB**. **1** and **2** were synthesized according to literature.[1-2]

Ir(2-(2,4-difluorophenyl)-5-(trifluoromethyl)pyridine)$_2$(**1**) – **(IrF-XB)**

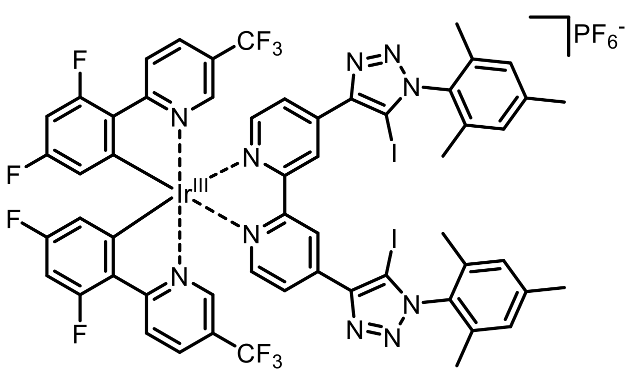

82 mg **1** (0.11 mmol) and 78 mg **2** (0.05 mmol) were placed in a microwave vial and the vial was sealed. After threefold evacuation and refilling with nitrogen, 20 mL CH_2Cl_2 was added. The suspension was then heated to 70 °C for 17 h. During that time, the material slowly dissolved due to the reaction. Afterwards, the reaction mixture cooled down to ambient temperature and 100 mL CH_2Cl_2 were added. The organic phase was washed two

times with aqueous NH_4PF_6 solution and with water. Subsequently, the organic phase was dried over Na_2SO_4 and the solvent was removed *in vacuo* to obtain the crude product. 43 mg (0.03 mmol, 25%) of the product in sufficient purity were obtained *via* column chromatography (silica, 15% ethyl acetate in CH_2Cl_2) by combination of the purest fractions.

^{1}H NMR (CD_2Cl_2, 300 MHz): δ = 1.92 (s, 6H), 1.94 (s, 6H), 2.42 (s, 6H), 5.81 (dd, 8.1 Hz, 2.3 Hz, 2H), 6.70 – 8.80 (m, 2H), 7.12 (s, 4H), 7.80 (s, 2H), 8.08 – 8.19 (m, 4H), 8.48 – 8.60 (m, 4H), 9.50 (d, 1.6 Hz, 2H) ppm.

^{19}F NMR (CD_2Cl_2, 470 MHz): δ = –103.8 (dt, 12.7 Hz, 2.9 Hz, 2F), –100.5 – –100.4 (m, 2F), –73.2 (d, 711.1 Hz, 6F), –63.6 (s, 6F) ppm.

HR-ESI-MS (ESI-TOF) m/z: $[M+Na]^+$ calcd. for $C_{56}H_{38}F_{10}I_2IrN_{10}$, 1487.0834; found, 1487.0861. Error:
-1.5 ppm.

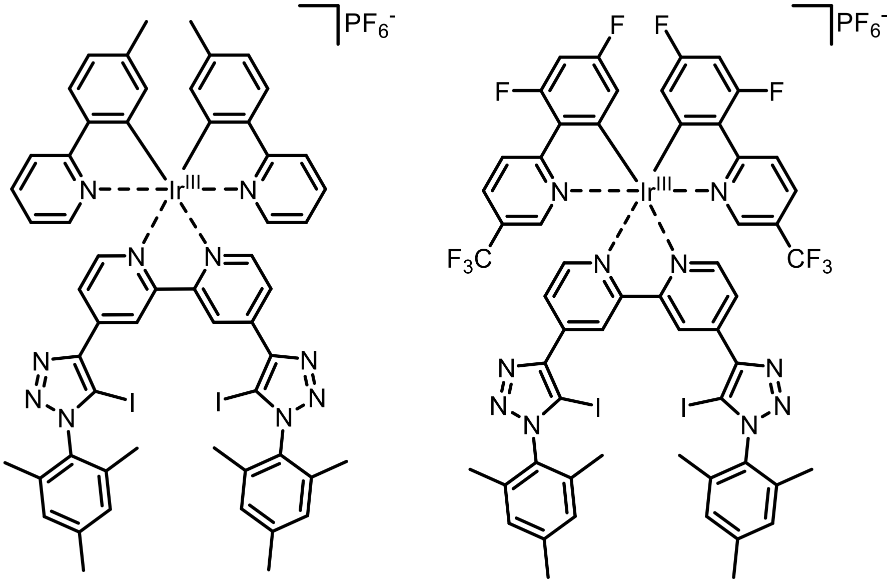

Scheme S2. Direct comparison of the previously published Ir(III)-based anion sensor (left)[1] and the improved system (right).

NMR spectroscopy

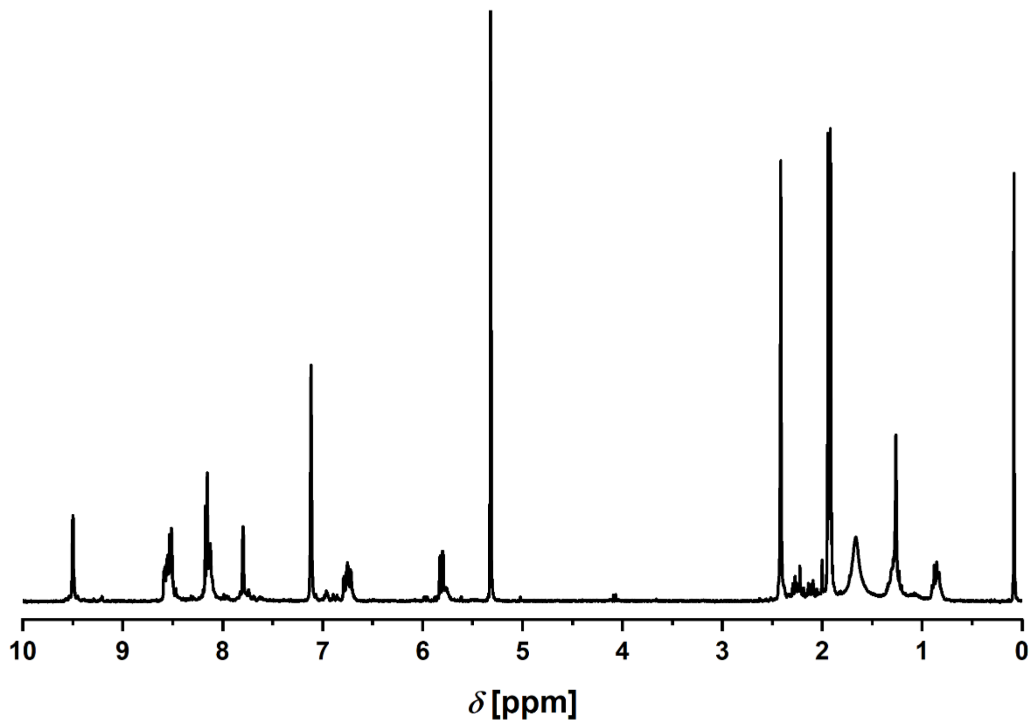

Figure S1. ^{1}H NMR spectrum of **IrF-XB** in CD_2Cl_2 at 300 MHz.

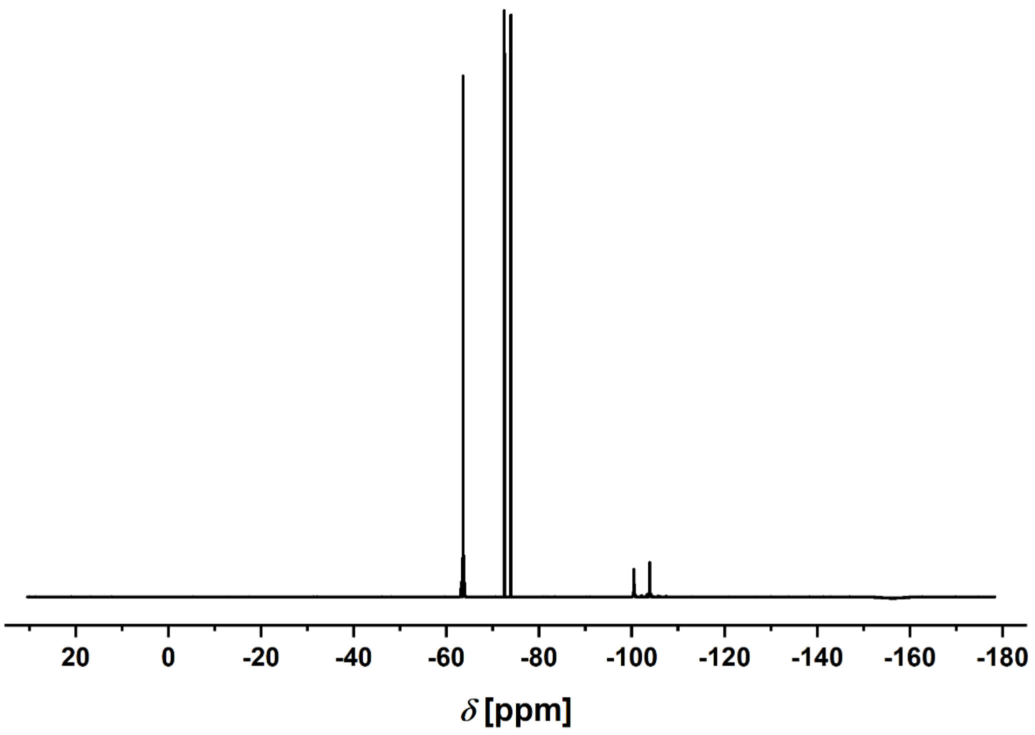

Figure S2. ^{19}F NMR spectrum of **IrF-XB** in CD_2Cl_2 at 470 MHz.

Emission titrations of IrF-XB

The emission titration experiments were performed at 23 °C in air saturated acetonitrile containing **IrF-XB** (c = 5·10^{-6} mol L^{-1}, host, titrant) and the anions (Cl^-, Br^-, PF_6^-, OAc^-, guest) were added as tetrabutylammonium salt solution in acetonitrile with **IrF-XB** (c = 5·10^{-6} mol L^{-1}). The emission maxima were determined *via* a Gauss fit of the emission bands. That approach turned out to estimate the precise maximum of the emission in a very simple manner and, subsequently, yielded robust fits with Bindfit. This open-source tool accessible at supramolecular.org, was applied for data evaluation and fitting.[4] The given errors are asymptotic error values at the 95% confidence interval level. For detailed information see a literature report.[5]

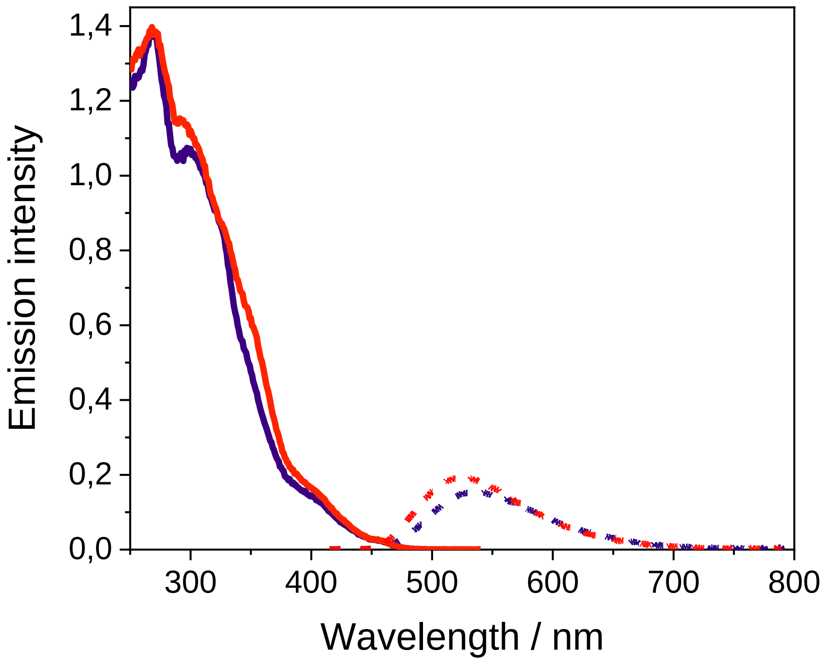

Figure S3. Steady state excitation (solid lines, λ_{em} = 550 nm) and emission spectra (dashed lines, λ_{ex} = 400 nm) of IrF-XB in aerated acetonitrile (blue) and upon addition of 20 eq. of tetrabutylammonium chloride (red).

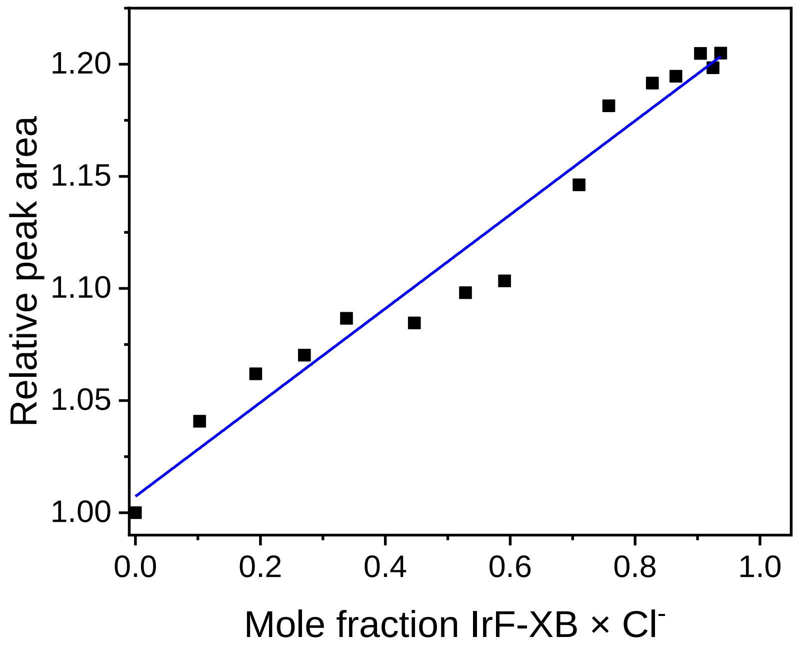

Figure S4. Relative peak area plotted against the mole fraction of **IrF-XB × Cl**$^-$ (black). The values of the relative peak area were calculated by integration of the spectra obtained in the titration of **IrF-XB** against chloride. Mole fractions were calculated from the fit depicted as **Fig 2**. The linear fit of the data (blue, Intercept = 1.0073 ± 0.0074, slope = 0.2094 ± 0.0116) was used to estimate the relative emission quantum yield of pure **IrF-XB × Cl**$^-$ of 1.2167 compared to **IrF-XB**.

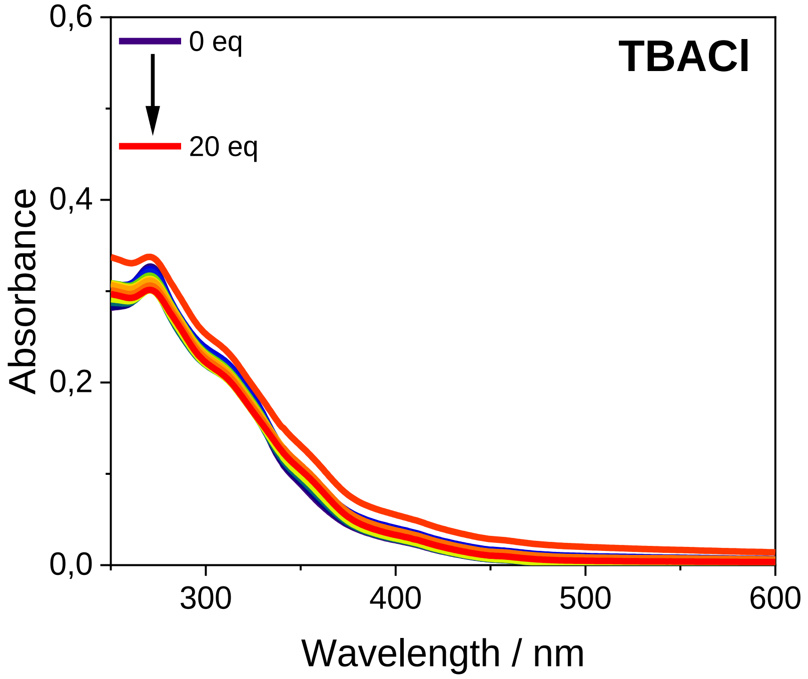

Figure S5. UV/vis spectra obtained in the titration experiment of **IrF-XB** (5×10^{-6} M^{-1} in aerated acetonitrile) against tetrabutylammonium chloride.

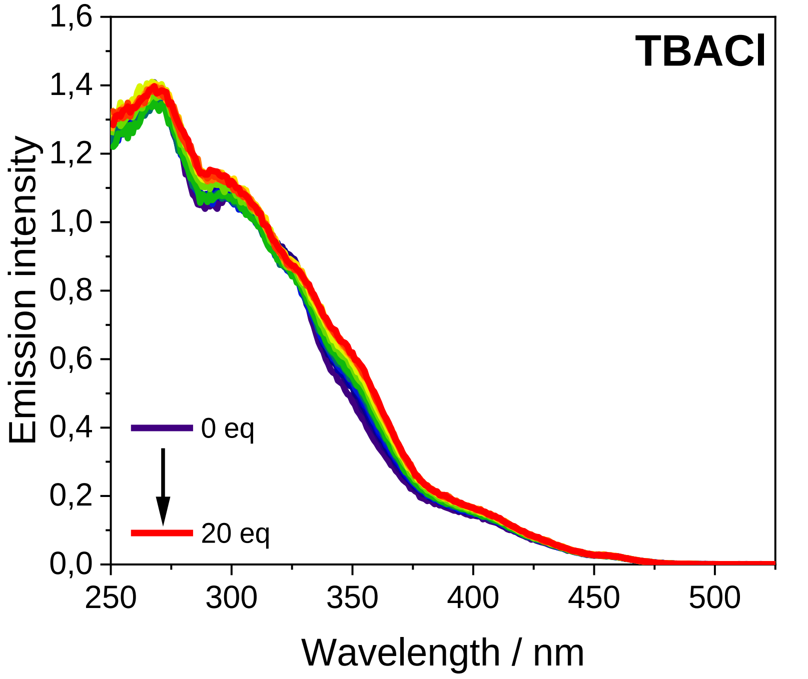

Figure S6. Excitation spectra obtained in the titration experiment of **IrF-XB** (5×10^{-6} M^{-1} in aerated acetonitrile, λ_{em} = 550 nm) against tetrabutylammonium chloride.

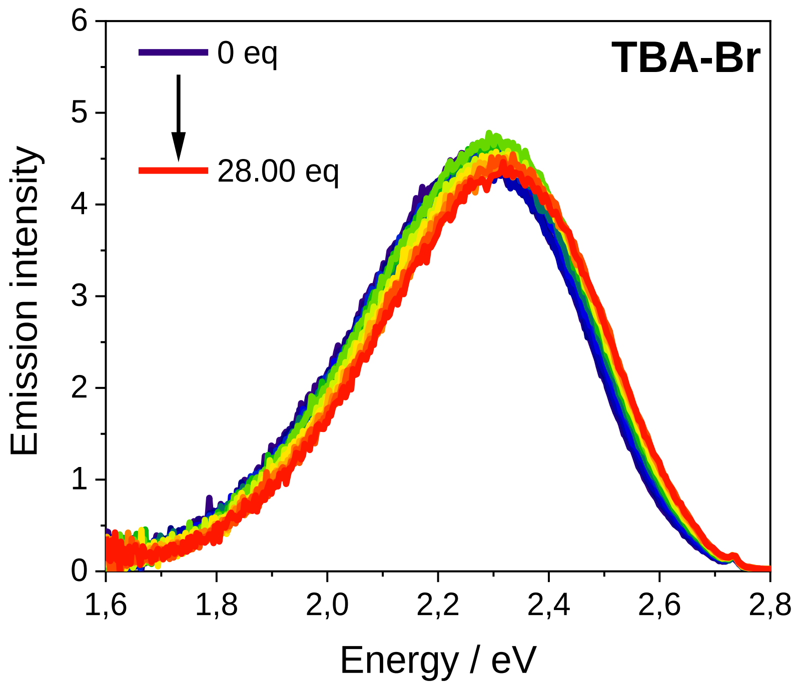

Figure S7. Emission spectra obtained in the titration experiment of **IrF-XB** (5×10^{-6} M^{-1} in aerated acetonitrile, λ_{ex} = 400 nm) against tetrabutylammonium bromide.

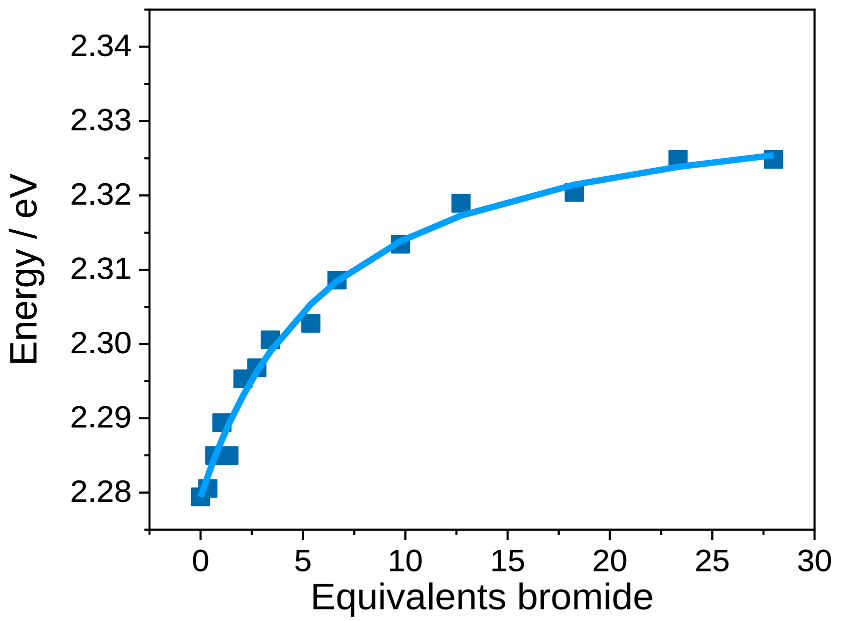

Figure S8. Fit of the emission maxima (in eV) against the guest equivalents added during the titration of **IrF-XB** with tetrabutylammonium bromide (K_a = 3.4 $\times 10^4$ M^{-1}).

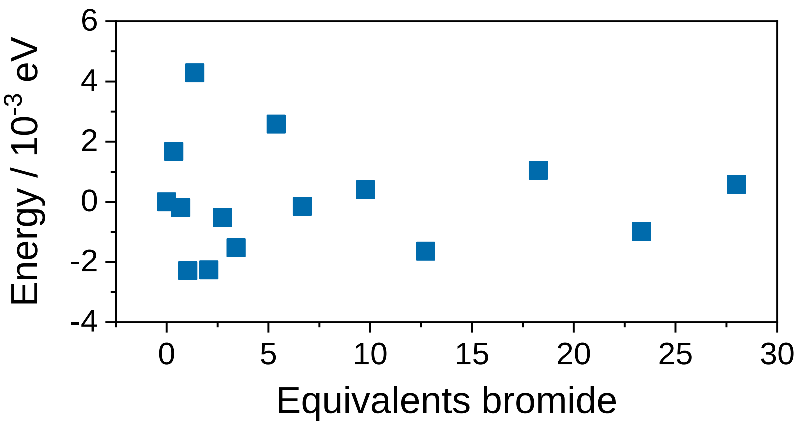

Figure S9. Residuals obtained by the fit of the emission maxima during the titration of **IrF-XB** against tetrabutylammonium bromide.

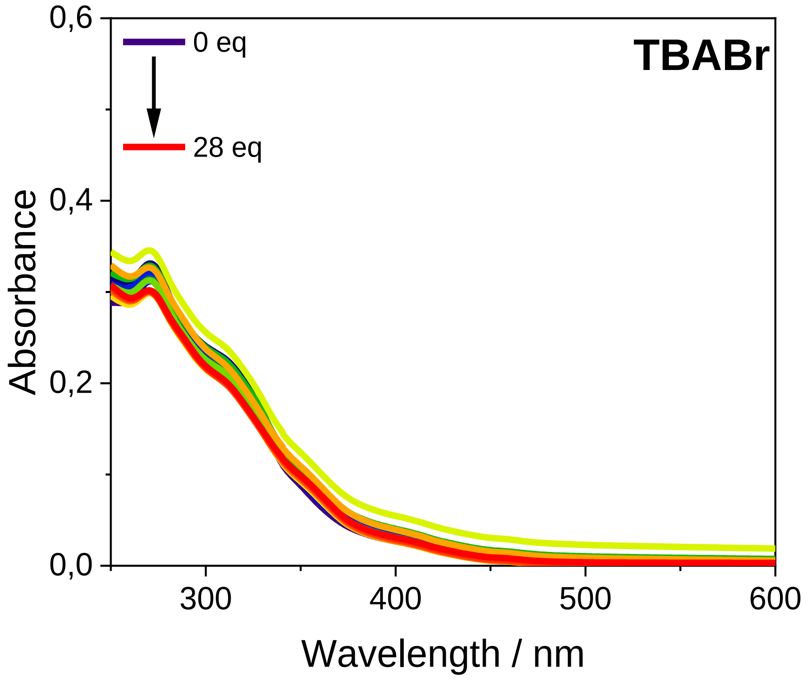

Figure S10. UV/vis spectra obtained in the titration experiment of **IrF-XB** (5×10^{-6} M^{-1} in aerated acetonitrile) against tetrabutylammonium bromide.

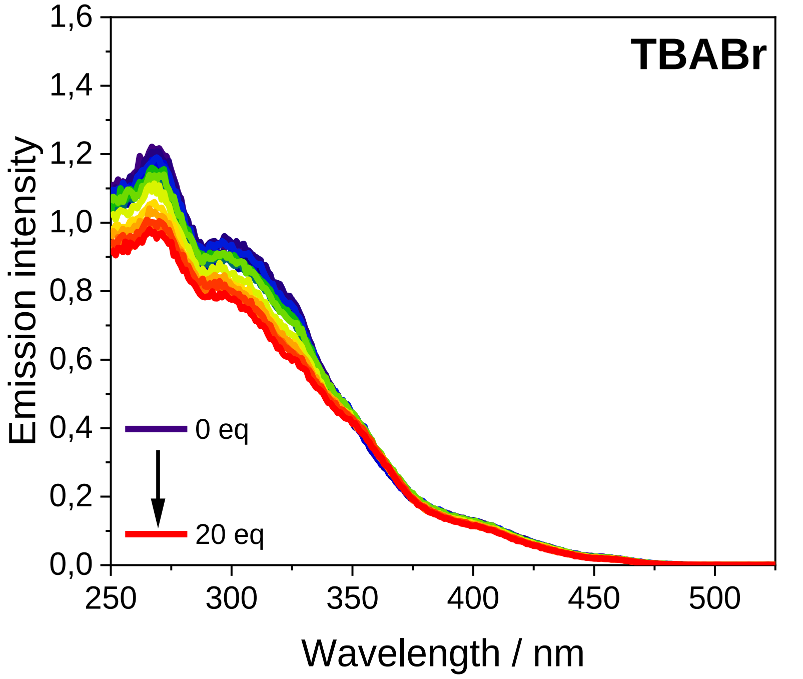

Figure S11. Excitation spectra obtained in the titration experiment of **IrF-XB** (5×10^{-6} M^{-1} in aerated acetonitrile, λ_{em} = 550 nm) against tetrabutylammonium bromide.

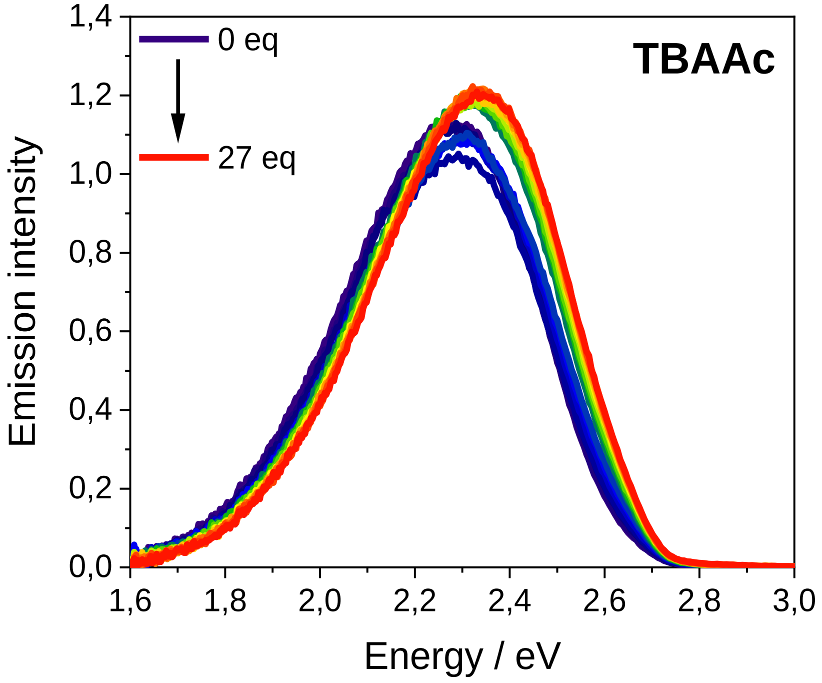

Figure S12. Emission spectra obtained in the titration experiment of **IrF-XB** (5×10^{-6} M^{-1} in aerated acetonitrile, λ_{ex} = 400 nm) against tetrabutylammonium acetate.

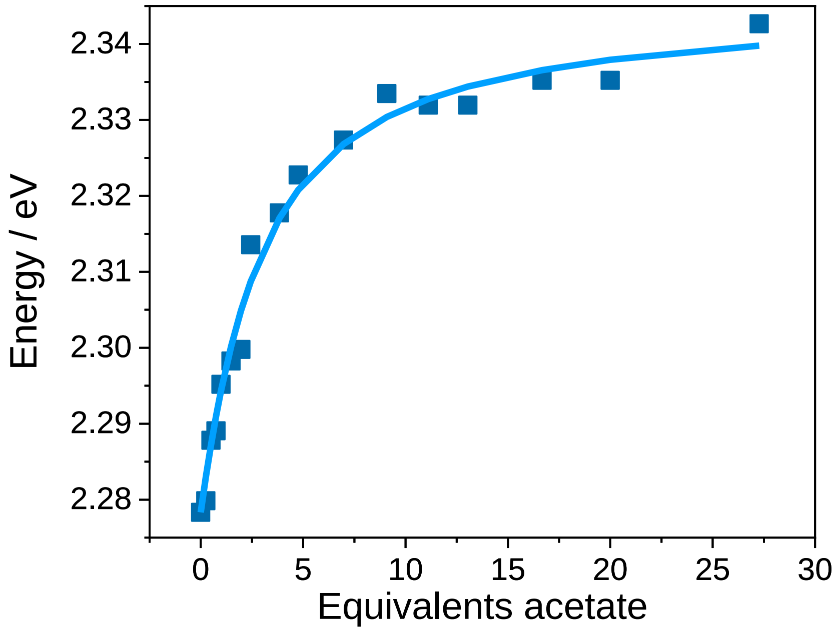

Figure S13. Fit of the emission maxima (in eV) against the guest equivalents added during the titration of **IrF-XB** with tetrabutylammonium acetate (K_a = 8.4 ×10^4 M^{-1}).

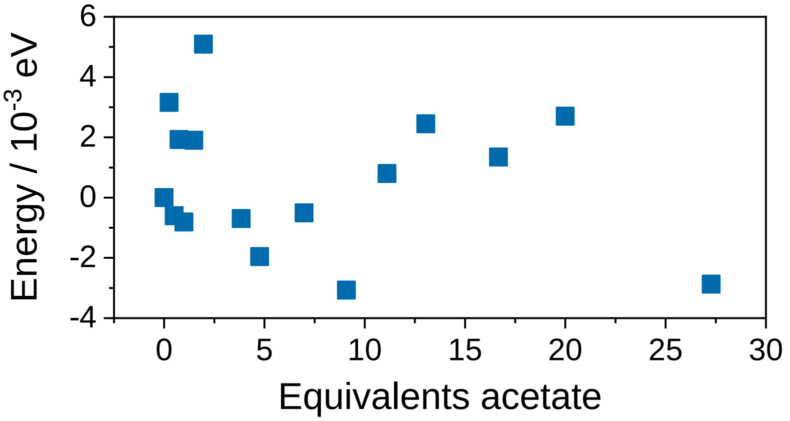

Figure S14. Residuals obtained by the fit of the emission maxima during the titration of **IrF-XB** against tetrabutylammonium acetate.

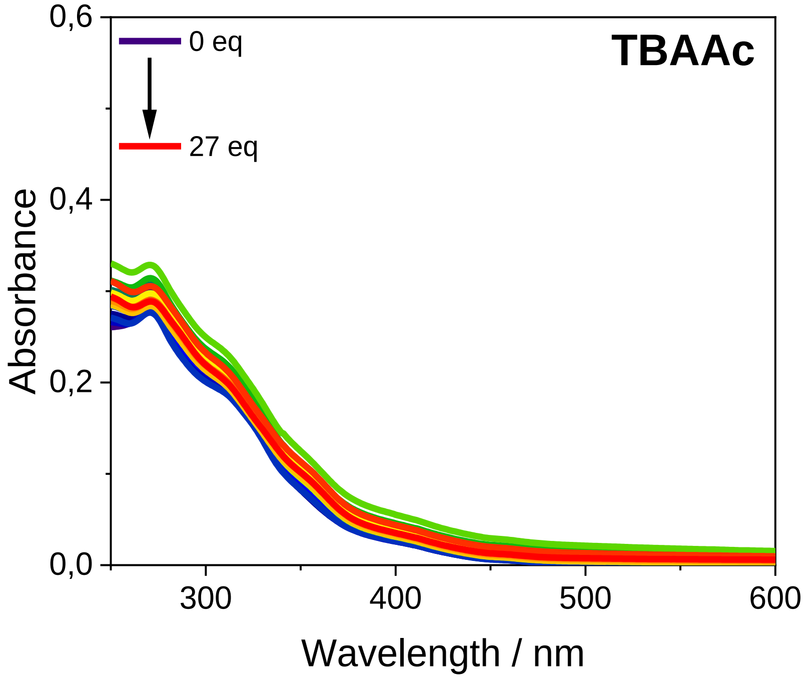

Figure S15. UV/vis spectra obtained in the titration experiment of **IrF-XB** (5×10^{-6} M^{-1} in aerated acetonitrile) against tetrabutylammonium acetate.

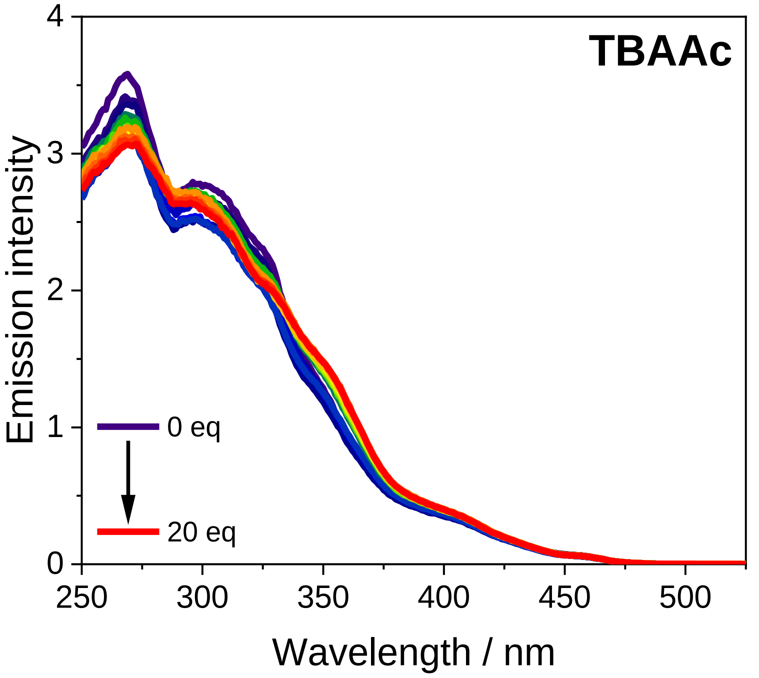

Figure S16. Excitation spectra obtained in the titration experiment of **IrF-XB** (5×10^{-6} M^{-1} in aerated acetonitrile, λ_{em} = 540 nm) against tetrabutylammonium acetate.

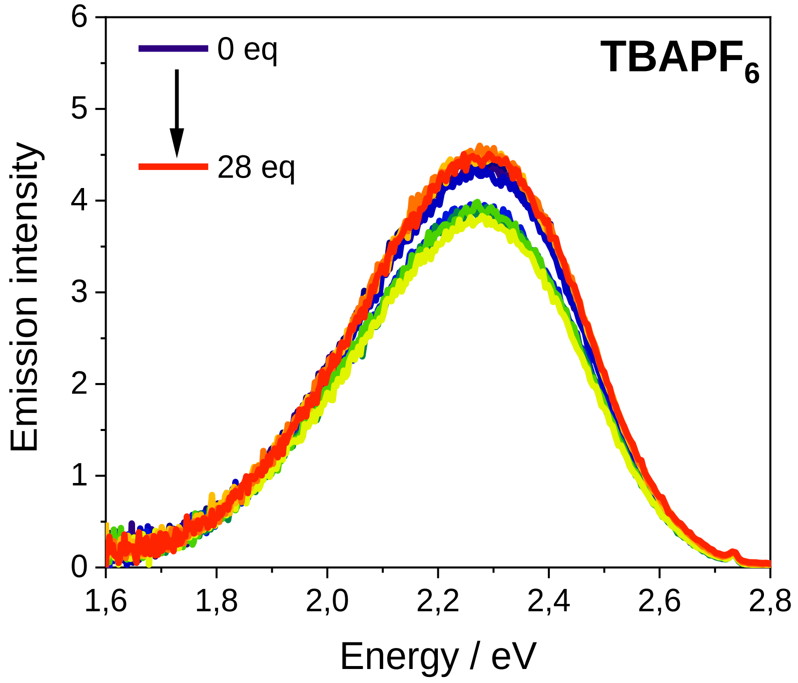

Figure S17. Emission spectra obtained in the titration experiment of **IrF-XB** (5×10^{-6} M^{-1} in aerated acetonitrile, λ_{ex} = 400 nm) against tetrabutylammonium hexafluorophosphate.

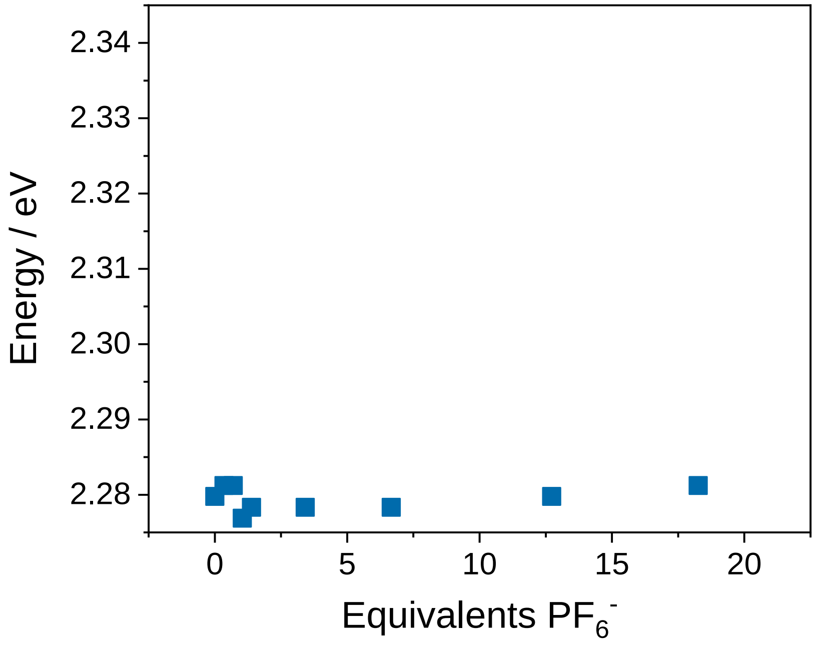

Figure S18. Progress of the emission maximum during the titration of **IrF-XB** with tetrabutylammonium hexafluorophosphate.

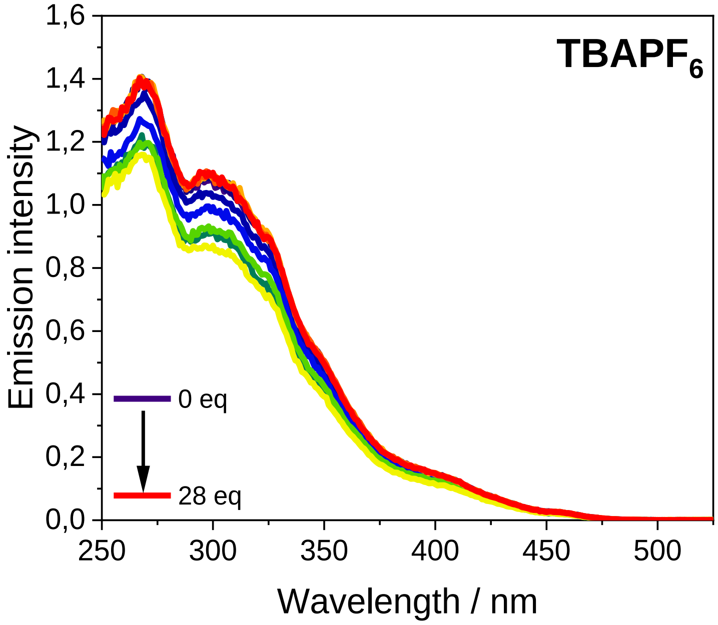

Figure S19. Excitation spectra obtained in the titration experiment of **IrF-XB** (5×10^{-6} M^{-1} in aerated acetonitrile, λ_{em} = 550 nm) against tetrabutylammonium hexafluorophosphate.

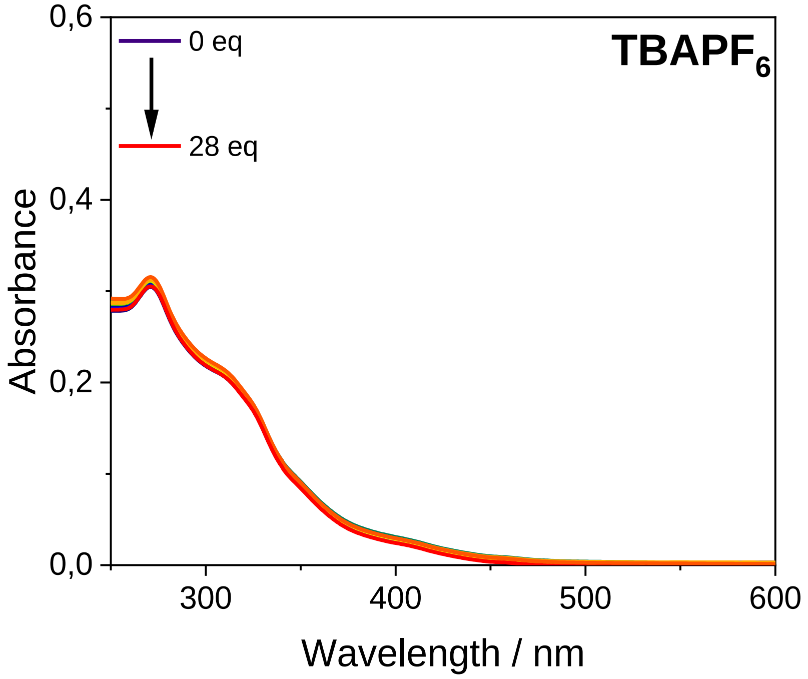

Figure S20. UV/vis spectra obtained in the titration experiment of **IrF-XB** (5×10^{-6} M^{-1} in aerated acetonitrile) against tetrabutylammonium hexafluorophosphate.

Table S1. Association constants of **IrF-XB** determined *via* emission titrations in 5×10^{-6} M acetonitrile solutions in comparison to the literature known complex. Data fitted using the 1 to 1 association model in Bindfit.[4]

		Cl^-	Br^-	AcO^-	PF_6^-
K_a (M^{-1})	This study	$1.6\times10^5 \pm 7$ %	$3.6\times10^4 \pm 11$ %	$8.4\times10^4 \pm 13$ %	–
	Reference [1]*	$5.7\times10^4 \pm 0.3$ %	$2.5\times10^4 \pm 0.3$ %	$2.2\times10^4 \pm 0.2$ %	–

*Constrained comparability due to different evaluation method used the assessment of the association constants.

Table S2. Overview of the spectral characteristics during the emission titrations.

	Rel. intensity change (end/start)	End eq. guest/Mole fraction HG	Maximum start/eV-	Maximum end/eV
**Chloride**	1.21	20.1 / 0.94	2.28409	2.33591
**Bromide**	0.95	28 / 0.83	2.27941	2.32483
**Acetate**	1.06	27.3 / 0.92	2.27833	2.34267
**Hexafluorophosphate**	1.02	28 / -	2.27977	2.28265

Computational details

All quantum chemical calculations addressing the ground state structures of **IrF-XB**, **IrF-XB × Cl**, **IrF-XB × Br**, **IrF-XB × Ac** and **(IrF-XB)$_2$ × Ac** were performed utilizing theGaussian16[6] program. Singlet (S_0) and triplet ground state (T_1) equilibrium structures and electronic properties of the Ir(III) complexes were obtained at the density functional level of theory (DFT) utilizing the B3LYP[7] exchange correlation (XC) functional. The def2-SVP[8] basis set as well as the respective core potentials were applied for all atoms. Vibrational analysis was carried out subsequently for the optimized ground state structure to verify that a minimum on the potential energy (hyper-)surface (PES) was obtained. Effects of interaction with acetonitrile solvent CH_3CN: ε=35.69, n=1.8069) were taken into account by the solute electron density (SMD) variant of the integral equation formalism of the polarizable continuum model (IEFPCM).[9] All calculations were performed including D3 dispersion correction with Becke-Johnson damping (D3BJ).[10]

Exemplarily, scalar relativistic effects as involved in the singlet-triplet population transfer by means of intersystem-crossing were explored for **IrF-XB** and **IrF-XB × Cl**. To evaluate scalar relativistic effects on the various excited states within the Franck-Condon region, calculations were performed utilizing ORCA 5.0.2,[11] *i.e.*, employing the scalar relativistic zeroth-order regular approximation (SR-ZORA).[12] TDDFT calculations were performed using the B3LYP/G XC functional.[13] The SARC-ZORA-TZVP[14] basis set was utilized for Ir and I, while all other atoms were described using the respective def2-TZVP basis sets (with the corresponding SARC/J auxiliary basis set).[15] The 50 lowest singlet-singlet and singlet-triplet excitations (spin-free, SF, states) were calculated, while the spin-orbit couplings (SOCs) between these states and the singlet ground state were obtained at the SR-ZORA-TDDFT level of theory – yielding the respective spin-orbit (SO) states. Implicit solvent effects (CH_3CN) were taken into consideration with the conductor-like polarizable continuum model (CPCM)[16].

Simulated Franck-Condon photophysics and intersystem-crossing

Insight regarding the nature of the electronic transitions underlying the absorption features of **IrF-XB** and **IrF-XB × Cl** was obtained at the (scalar-relativistic) time-dependent density functional level of theory (SR-TDDFT). The simulated electronic absorption spectra are depicted in **Figure S21**. In case of **IrF-XB** (**Figure S21 a)**), the performed SR-TDDFT simulations reveal mainly three excitations, *i.e.*, the two spin-orbit states SO_{25} and SO_{29} centred at 379 and 367 nm (3.27 and 3.38 eV), respectively, contributing to the shoulder absorption feature at approximately 380 nm as well as SO_{167} (at 293 nm, 4.23 eV) corresponding to the main absorption feature at ~280 nm. Noteworthy, these three spin-orbit states (SO_{25}, SO_{29} and SO_{167}) feature only marginal triplet state

contributions and, thus, are mainly of singlet character (see Table S1) and involve ligand-to-ligand charge transfer (^{1}LLCT), intra-ligand charge transfer (^{1}ILCT) and metal-to-ligand charge transfer (^{1}MLCT) transitions. In addition and typical for such Ir(III) complexes, the visible-light absorption in the range 440 to 470 nm reveals a weak absorbing formally spin-forbidden $^{1/3}$MLCT transition shoulder.[17] In case of **IrF-XB** this shoulder is described by means of an excitation into SO_{14} (at 417 nm, 2.97 eV) – a mixed $^{1/3}$MLCT state involving all three co-ligands. The underlying spin-free states, *e.g.*, S_8 and T_7, feature a pronounced spin-orbit coupling (SOC) of up to nearly 450 cm^{-1} (**Tables S2** and **S5**).

In case of **IrF-XB × Cl**, almost identical excited state properties were predicted by SR-TDDFT within the fully relaxed singlet ground state structure (**Figure S21 b)** as well as **Table S4** and **S5**). Therefore, the simulations reveal that the chlorine anion binding does not significantly alter the energetics of electronic transitions involved in the light-activation of the chemo-sensor.

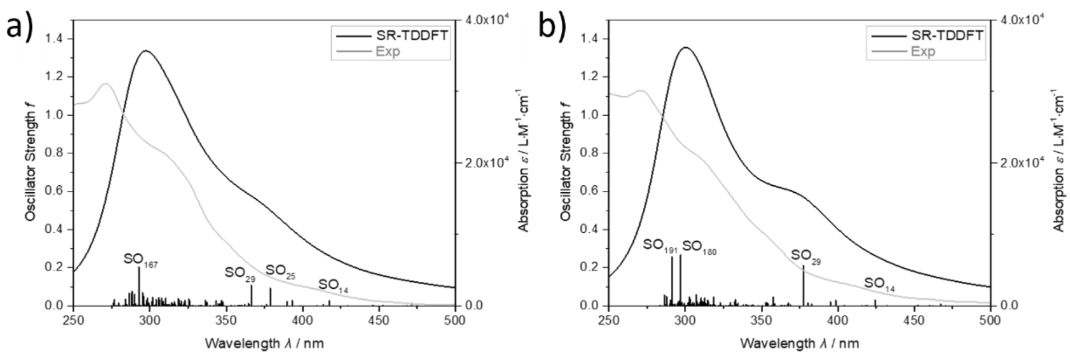

Figure S21. a) Simulated (black line) and experimental (grey line) absorption spectra of **IrF-XB** and b) **IrF-XB × Cl** obtained by SR-TDDFT; solvent effects (CH_3CN) were incorporated by means of CPCM. Simulated transitions are broadened by Lorentzian functions with a full width at half maximum of 0.25 eV.

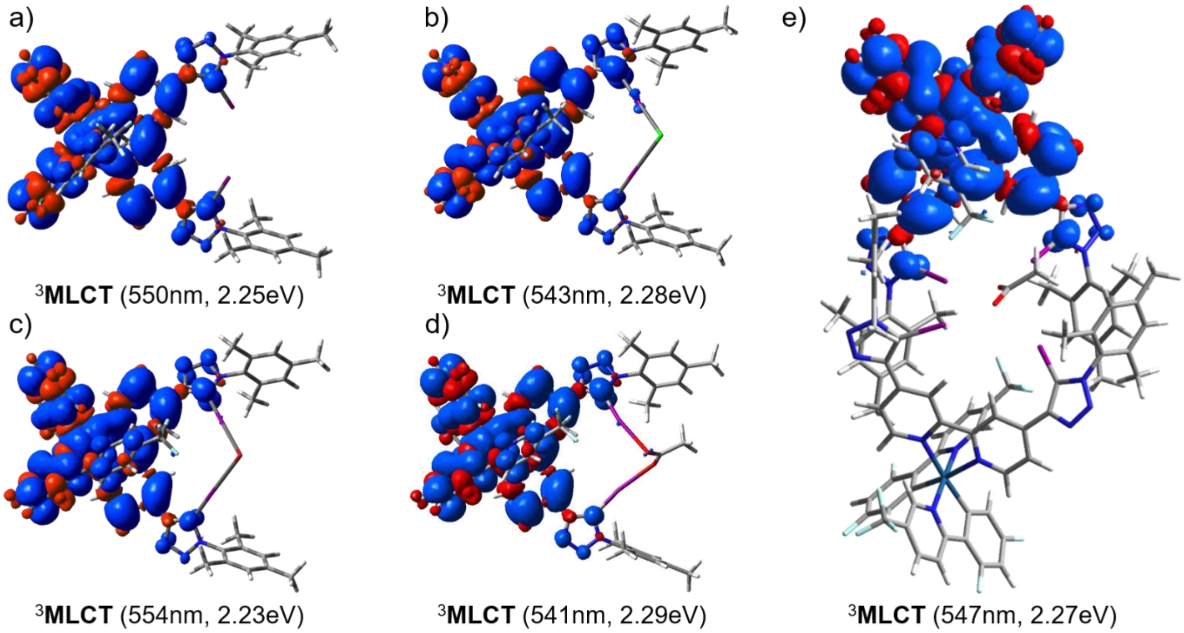

Figure S22. Spin density of the lowest energy triplet state of a) **IrF-XB**, b) **IrF-XB × Cl**, c) **IrF-XB × Br**, d) **IrF-XB × Ac** and e) **(IrF-XB)$_2$ × Ac** within their fully relaxed equilibrium structures as obtained at the DFT level of theory. Electronic characters as well as emission wavelengths and energies are indicated as predicted based on TDDFT (spin-forbidden singlet-triplet excitations).

Table S3. Calculated energies, oscillator strengths and composition of the lowest spin-orbit states in **IrF-XB** in CH_3CN based on the spin-free singlet and triplet states, respectively.

State	Composition	Weight / %	Δ*E* / eV	λ / nm	*f*
SO_{14}	S_2	51	2.97	417	0.0274
	T_5	18			
	T_7	8			
SO_{25}	S_5	38	3.27	379	0.0920
	S_8	48			
SO_{29}	S_5	40	3.38	367	0.1086
	S_8	38			
SO_{167}	S_{29}	13	4.23	293	0.2027
	S_{33}	44			
	S_{42}	8			

Table S4. Simulated excited state properties of the low-lying singlet-singlet and singlet-triplet excitations of **IrF-XB** in CH_3CN such as excitation energies (in eV), excitation wavelengths (in nm), oscillator strengths, spin contamination and leading transitions as represented by charge density differences (CDDs; charge transfer takes place from red to blue).

State	ΔE / eV	λ / nm	f	$\langle s^2 \rangle$	Character
S_2	3.04	408	0.0661	0.000	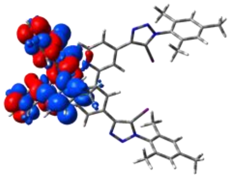
S_5	3.28	378	0.2824	0.000	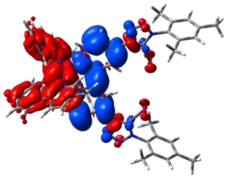
S_8	3.59	310	0.1051	0.000	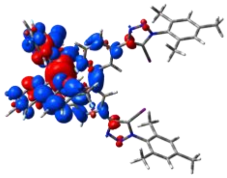
S_{29}	4.08	299	0.2094	0.000	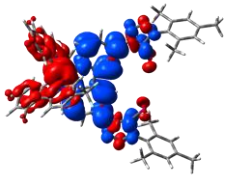
S_{33}	4.18	297	0.1752	0.000	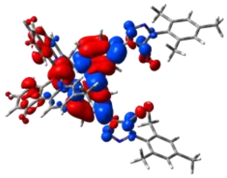
S_{42}	4.35	295	0.3187	0.000	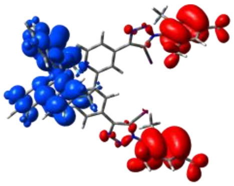
T_5	2.91		0.000	2.000	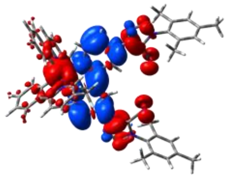
T_7	3.10		0.000	2.000	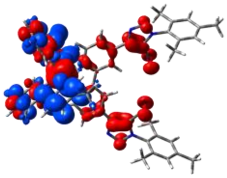

Table S5. Calculated energies, oscillator strengths and composition of the lowest spin-orbit states in **IrF-XB × Cl** in CH_3CN based on the spin-free singlet and triplet states, respectively.

State	Composition	Weight / %	ΔE / eV	λ / nm	f
SO_{14}	S_2	62	2.92	424	0.0294
	T_6	9			
	T_7	9			
SO_{29}	S_5	66	3.29	377	0.2130
	S_{11}	9			
SO_{180}	S_{36}	65	4.18	297	0.2658
	S_{37}	15			
SO_{191}	S_{39}	46	4.26	291	0.2566
	S_{41}	11			
	S_{42}	26			

Table S6. Simulated excited state properties of the low-lying singlet-singlet and singlet-triplet excitations of **IrF-XB × Cl** in CH_3CN such as excitation energies (in eV), excitation wavelengths (in nm), oscillator strengths, spin contamination and leading transitions as represented by charge density differences (CDDs; charge transfer takes place from red to blue).

State	Δ*E* / eV	λ / nm	*f*	⟨s²⟩	Character
S_2	3.03	410	0.062	0.000	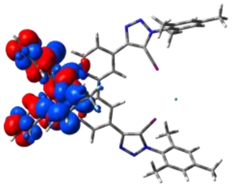
S_5	3.30	376	0.341	0.000	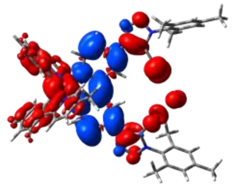
S_{11}	3.65	340	0.076	0.000	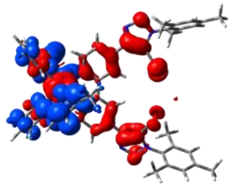
S_{36}	4.15	299	0.209	0.000	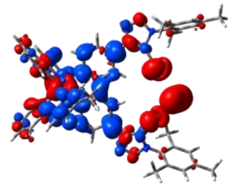
S_{37}	4.16	298	0.175	0.000	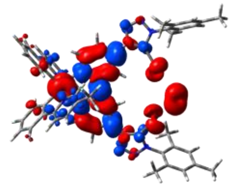
S_{39}	4.21	295	0.319	0.000	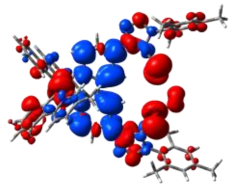
S_{41}	4.22	294	0.017	0.000	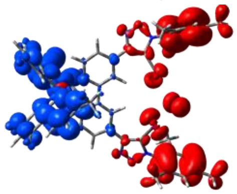
S_{42}	4.25	292	0.002	0.000	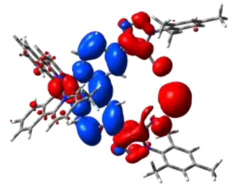

State	ΔE / eV	λ / nm	f	⟨s^2⟩	Character
T_6	3.09	401	0.000	2.000	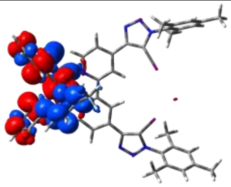
T_7	3.10	400	0.000	2.000	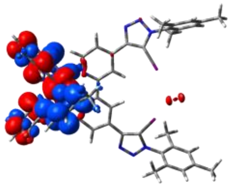

Table S7. Spin-orbit couplings ($\langle T_j|\hat{H}_{SOC}|S_i\rangle$) in cm^{-1} for **IrF-XB** and **IrF-XB × Cl** in CH_3CN.

IrF-XB	T_5 (^{3}MLCT)	T_7 (^{3}MLCT)	IrF-XB × Cl	T_6 (^{3}MLCT)	T_7 (^{3}MLCT)
**S_2 (^{1}MLCT)**	100	364	**S_2 (^{1}MLCT)**	395	382
**S_5 (^{1}MLCT/^{1}ILCT)**	21	156	**S_5 (^{1}MLCT/^{1}ILCT)**	156	157
**S_8 (^{1}MLCT)**	175	447	**S_{11} (^{1}MLCT/^{1}ILCT)**	255	530
**S_{29} (^{1}MLCT/^{1}ILCT)**	91	82	**S_{36} (^{1}MLCT/^{1}ILCT)**	60	132
**S_{33} (^{1}MLCT/^{1}ILCT)**	154	41	**S_{37} (^{1}MLCT/^{1}ILCT)**	57	98
**S_{42} (^{1}LLCT)**	10	8	**S_{39} (^{1}MLCT/^{1}ILCT)**	55	116
			**S_{41} (^{1}LLCT)**	100	18
			**S_{42} (^{1}MLCT/^{1}ILCT)**	29	22

Table S8. Bond length of C-I (d_1 and d_2) in angstrom, angle of C-I-Anion (θ_1 and θ_2) and prominent dihedral angels (δ_1 and δ_2) in **IrF-XB**, **IrF-XB × Cl**, **IrF-XB × Br**, **IrF-XB × Ac** and **(IrF-XB)$_2$ × Ac** as obtained within the singlet ground (S_0) and triplet ground state (T_1) equilibria, respectively.

	Å / °	S_0	T_1
**IrF-XB** 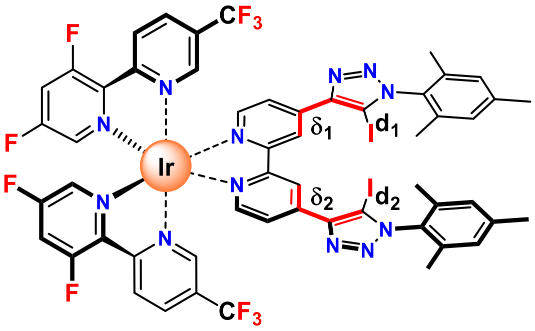	d_1	2.0786	2.0794
	d_2	2.0785	2.0793
	δ_1	15.0	15.4
	δ_2	15.8	15.7
**IrF-XB × Cl** 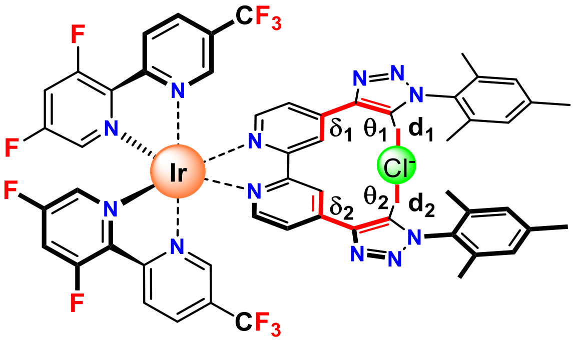	d_1	2.1265	2.1227
	d_2	2.1254	2.1233
	θ_1	178.3	178.6
	θ_2	180.0	178.5
	δ_1	–25.1	–4.1
	δ_2	21.4	22.1

**IrF-XB × Br**<br>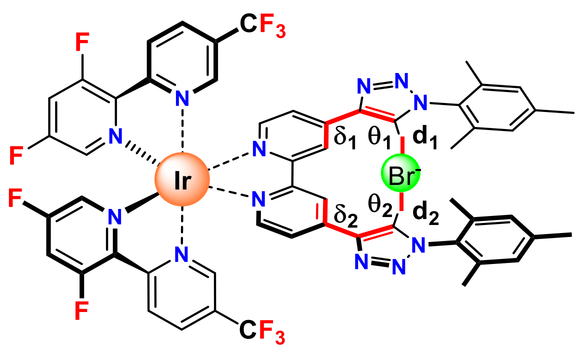	d_1	2.1202	2.1182
	d_2	2.1209	2.1189
	Θ_1	176.9	177.8
	Θ_2	177.1	178.3
	δ_1	–25.8	–28.2
	δ_2	23.5	29.0
**IrF-XB × Acetate**<br>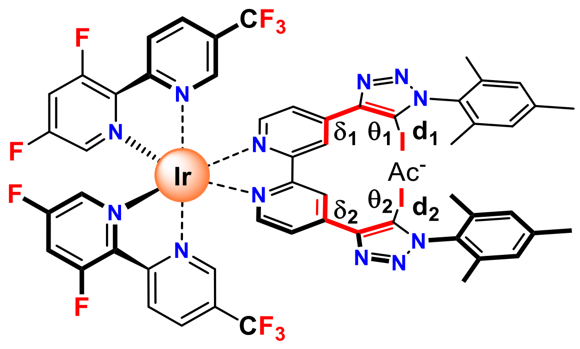	d_1	2.0958	2.0937
	d_2	2.1333	2.1326
	Θ_1	162.3	163.3
	Θ_2	170.3	170.2
	δ_1	-23.5	-27.5
	δ_2	-0.7	9.8
**$(IrF\text{-}XB)_2$ × Acetate**<br>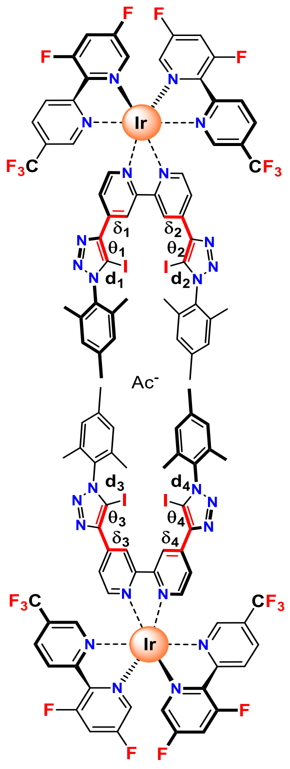	d_1	2.0922	2.0911
	d_2	2.1074	2.1044
	d_3	2.1247	2.1267
	d_4	2.0752	2.0753
	Θ_1	166.1	166.2
	Θ_2	177.1	177.4
	Θ_3	174.7	174.9
	Θ_4	102.0	101.6
	δ_1	34.4	34.5
	δ_2	-41.3	-38.4
	δ_3	3.2	2.9
	δ_4	-40.6	-40.6

References

[1] R. Kampes, R. Tepper, H. Görls, P. Bellstedt, M. Jäger, U. S. Schubert, *Chem. Eur. J.* **2020**, *26*, 14679-14687.

[2] M. S. Lowry, J. I. Goldsmith, J. D. Slinker, R. Rohl, R. A. Pascal, G. G. Malliaras, S. Bernhard, *Chem. Mater.* **2005**, *17*, 5712-5719.

[3] a) G. A. Reynolds, K. H. Drexhage, *Opt. Commun.* **1975**, *13*, 222-225; b) A. M. Brouwer, *Pure Appl. Chem.* **2011**, *83*, 2213-2228.

[4] http://supramolecular.org, accessed 19.04.2023.

[5] P. Thordarson, *Chem. Soc. Rev.* **2011**, *40*, 1305-1323.

[6] M. Frisch, G. Trucks, H. Schlegel, G. Scuseria, M. Robb, J. Cheeseman, G. Scalmani, V. Barone, G. Petersson, H. Nakatsuji, *Gaussian 16 Rev. B. 01, Wallingford, CT*, **2016**.

[7] a) A. D. Beck, *J. Chem. Phys.* **1993**, *98*, 5648-5652; b) C. Lee, W. Yang, R. G. Parr, *Phys. Rev. B* **1988**, *37*, 785-789.

[8] a) F. Weigend, *Physical chemistry chemical physics* **2006**, *8*, 1057-1065; b) F. Weigend, R. Ahlrichs, *Physical Chemistry Chemical Physics* **2005**, *7*, 3297-3305.

[9] a) A. V. Marenich, C. J. Cramer, D. G. Truhlar, *J. Phys. Chem. b* **2009**, *113*, 6378-6396; b) B. Mennucci, C. Cappelli, C. A. Guido, R. Cammi, J. Tomasi, *J. Phys. Chem. A* **2009**, *113*, 3009-3020.

[10] S. Grimme, S. Ehrlich, L. Goerigk, *J. Comput. Chem.* **2011**, *32*, 1456-1465.

[11] F. Neese, F. Wennmohs, U. Becker, C. Riplinger, *J. Chem. Phys.* **2020**, *152*, 224108.

[12] E. van Lenthe, E.-J. Baerends, J. G. Snijders, *J. Chem. Phys.* **1994**, *101*, 9783-9792.

[13] A. R. Kovrizhina, E. I. Samorodova, A. I. Khlebnikov, *Molbank* **2021**, *2021*, M1299.

[14] L. M. Debefve, C. J. Pollock, *Physical Chemistry Chemical Physics* **2021**, *23*, 24780-24788.

[15] D. A. Pantazis, F. Neese, *Theor. Chem. Acc.* **2012**, *131*, 1-7.

[16] Y. Takano, K. Houk, *J. Chem. Theory Comput.* **2005**, *1*, 70-77.

[17] S. K. Seth, P. Purkayastha, *Eur. J. Inorg. Chem.* **2020**, *2020*, 2990-2997.
